# Supplementary material for: AhR and SHP regulate phosphatidylcholine and S-adenosylmethionine levels in the one-carbon cycle
Source: Nat Commun. 2018 Feb 7;9:540. doi: 10.1038/s41467-018-03060-y (PMC5803255; doi:10.1038/s41467-018-03060-y)
Supplement: Supplementary file 1 — Supplementary Information [file 41467_2018_3060_MOESM1_ESM.pdf]

Supplementary Information

Supplementary Figures

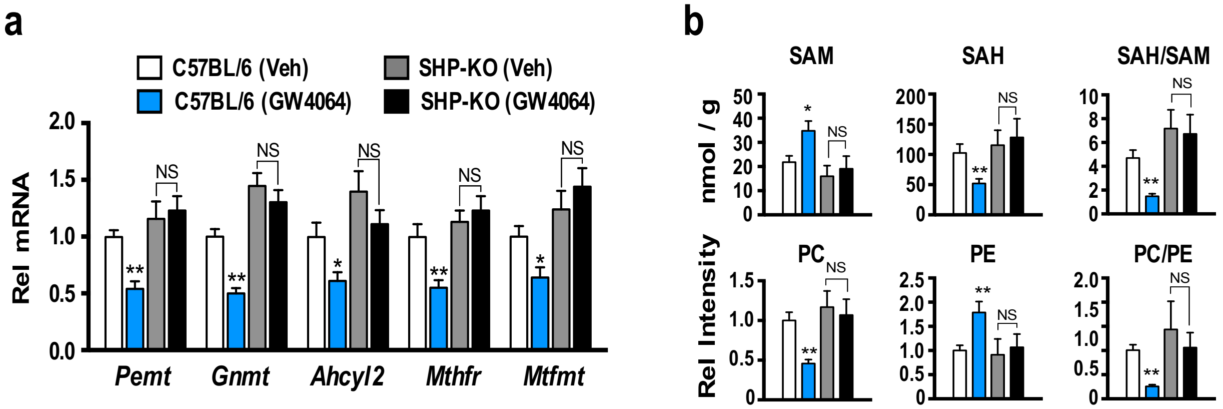

Supplementary Figure 1. (a-b) C57BL/6 mice and SHP-KO mice were fasted for 12 h and then, treated with GW4064 for 6 h. Livers were collected and mRNA levels of 1C cycle genes (a) or 1C cycle metabolites levels (b) were determined by q-RTPCR or LC-MS, respectively. Means +/- SD are shown (n=5 mice), and statistical significance was measured by two-way ANOVA with the FDR post-test and *P<0.05, **P<0.01, and NS, not statistically significant.

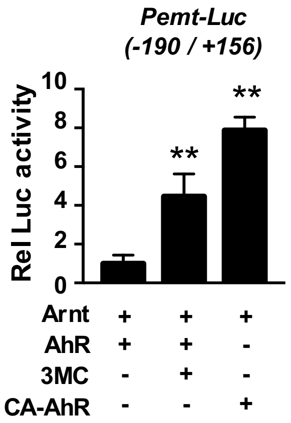

Supplementary Figure 2. Hepa1c1c7 cells were transfected with a *Pemt-luc* construct containing the WT AhR binding site in the *Pemt* promoter along with expression plasmids as indicated, treated with FGF19 for 2 h and luciferase activity was measured and normalized to β-galactosidase activity. Means +/- SD are shown (n=5), and statistical significance was measured using one-way ANOVA with the FDR post-test. *P<0.05, **P<0.01, and NS, not statistically significant.

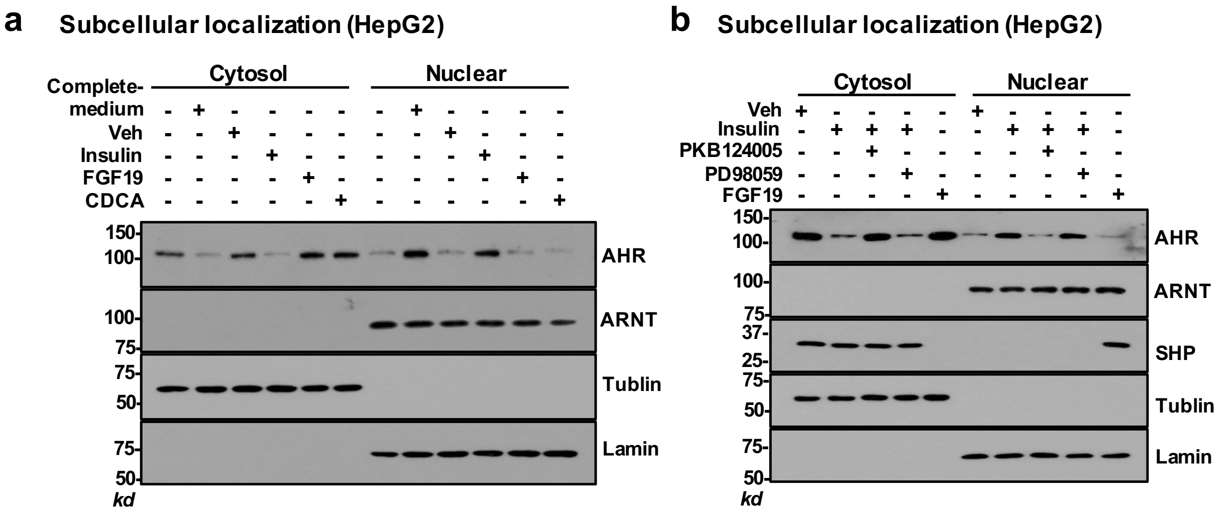

Supplementary Figure 3. (a) HepG2 cells were grown in low-glucose, serum-free media for 12 h and transferred for 15 min to complete medium or treated with insulin, FGF19, or CDCA for 15 min, then cells were harvested. (b) Cells were pre-treated with a PKB inhibitor, PKB124005, or an ERK inhibitor, PD98059, for 30 min prior to insulin treatment as indicated. Levels of AhR and SHP in the cytoplasmic and nuclear fractions were determined by IB. Full size immunoblots are in Supplementary Figure 13a.

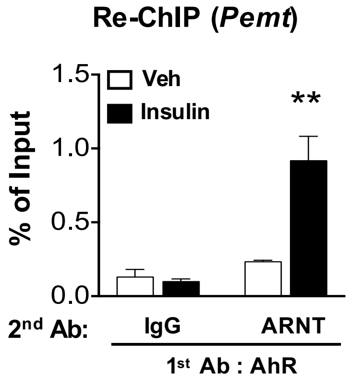

Supplementary Figure 4. Chromatin isolated from Hepa1c1c7 cells treated with insulin for 1 h was immunoprecipitated with AhR antibody, then eluted, and re-precipitated with ARNT antibody to examine the occupancy of ARNT in AhR-bound chromatin at the *Pemt* promoter. Means +/- SD are shown (n=5), and statistical significance was measured using two-way ANOVA with the FDR post-test. *P<0.05, **P<0.01, and NS, statistically not significant.

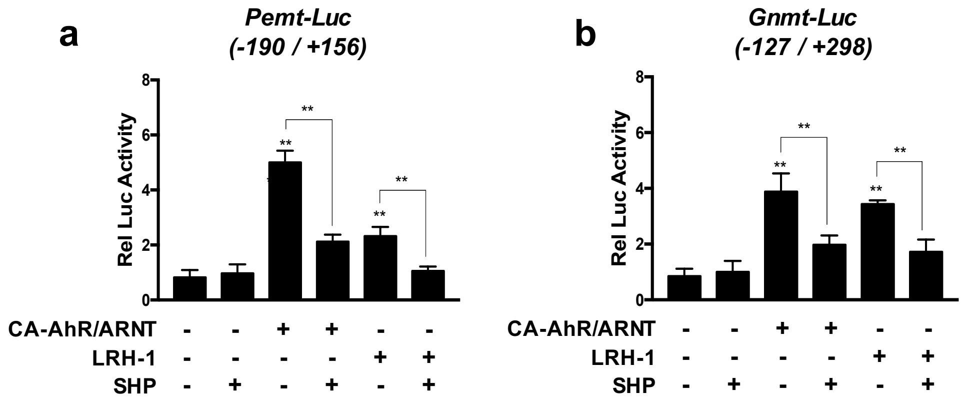

Supplementary Figure 5. Hepa1c1c7 cells were transfected with a *Pemt-luc* or *Gnmt-luc* promoter construct containing AhR binding sites along with expression plasmids as indicated. After 2 days, luciferase activities were measured. Means +/- SD are shown (n=5), and statistical significance was measured using one-way ANOVA with the FDR post-test. *P<0.05, **P<0.01, and NS, statistically not significant.

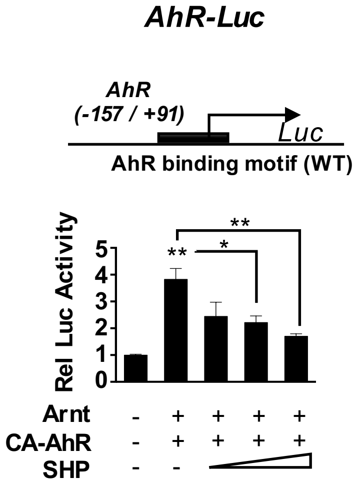

Supplementary Figure 6. Hepa1c1c7 cells were transfected with a *AhR-luc* construct containing the WT AhR binding site in the *AhR* promoter along with expression plasmids as indicated. After 2 days, cells were treated with FGF19 for 1 h, and luciferase activities were measured and normalized to β-galactosidase activity. Means +/- SD are shown (n=5), and statistical significance was measured using one-way ANOVA with the FDR post-test. *P<0.05, **P<0.01, and NS, statistically not significant.

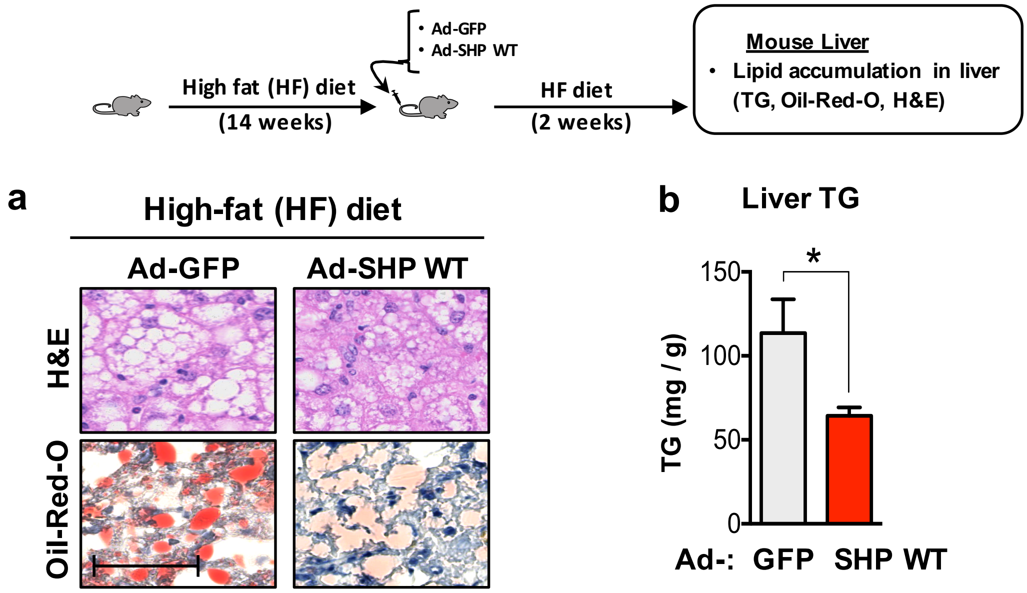

Supplementary Figure 7. Mice (n=5/group) were fed a high-fat diet for 14 weeks and Ad-GFP control or Ad-SHP were injected via the tail vein. Two weeks later (total = 16 weeks), livers were isolated. (a) Effects of SHP overexpression on lipid regulation in liver were determined by liver histology detected by Oil-Red-O staining and hematoxylin and eosin (H&E) staining. Scale bar: 100 μM (b) Triglyceride (TG) levels in liver were measured. Means +/- SD are shown (n=5 mice), and statistical significance was measured using by the Student's t-test. *P<0.05, **P<0.01, and NS, statistically not significant.

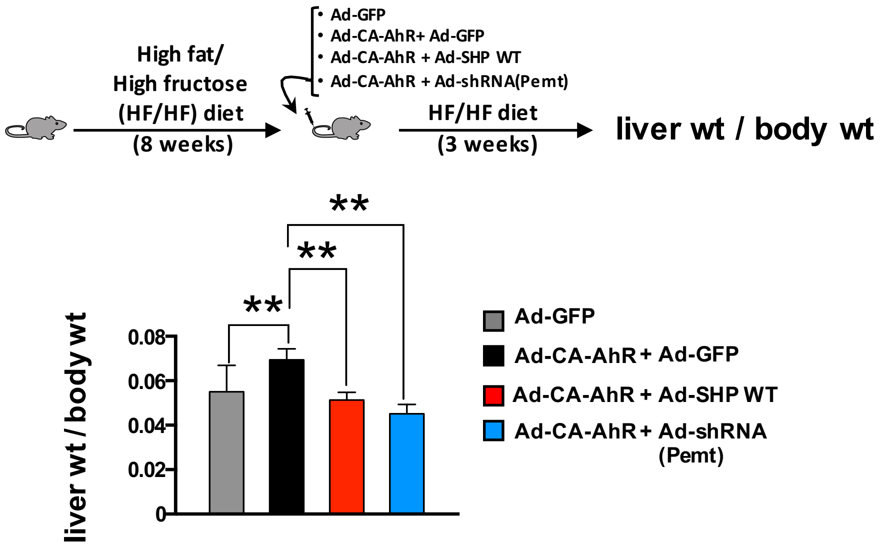

Supplementary Figure 8. Effects of expression of SHP or downregulation of *Pemt* on the CA-AhR-mediated increase in liver weight/body weight. Mice (n=5/group) were fed a HF/HF diet for 8 weeks, infected with the indicated adenovirus, and after 3 weeks, body and liver weights were measured. The ratio of liver weight/ body weight is plotted. Means +/- SD are shown (n=5 mice), and statistical significance was measured using one-way ANOVA with the FDR post-test. *P<0.05, **P<0.01, and NS, statistically not significant.

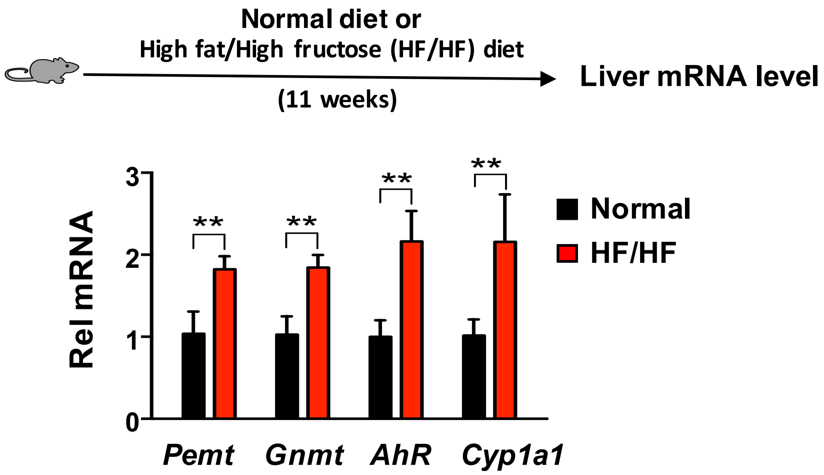

Supplementary Figure 9. Effects of a chronic HF/HF diet on hepatic expression of *Pemt, Gnmt, AhR, and Cyp1a1.* Mice (n=5/group) were fed either a normal chow diet (ND) or a high fat/high fructose (HF/HF) diet for 11 weeks, and then, mice were sacrificed and livers were collected. The mRNA levels of *Pemt, Gnmt, AhR, and Cyp1a1* were determined by q-RTPCR. Means +/- SD are shown (n=5 mice), and statistical significance was measured using the Student's t-test, *P<0.05, **P<0.01, and NS, statistically not significant.

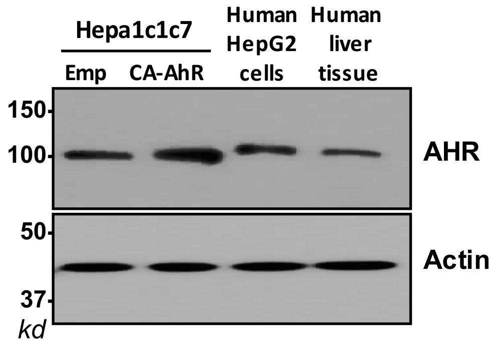

Supplementary Figure 10. Hepa1c1c7 cells were transfected with empty vector or pCMX-CA-AhR expressing human CA-AhR. Human HepG2 cells were also grown in normal media. After 2 days, protein was isolated from the cells as well as from human liver tissue and AhR or actin was detected by immunoblotting. As expected human CA-AhR and mouse WT AhR have similar mobilities. Full size immunoblots are in Supplementary Figure 13b.

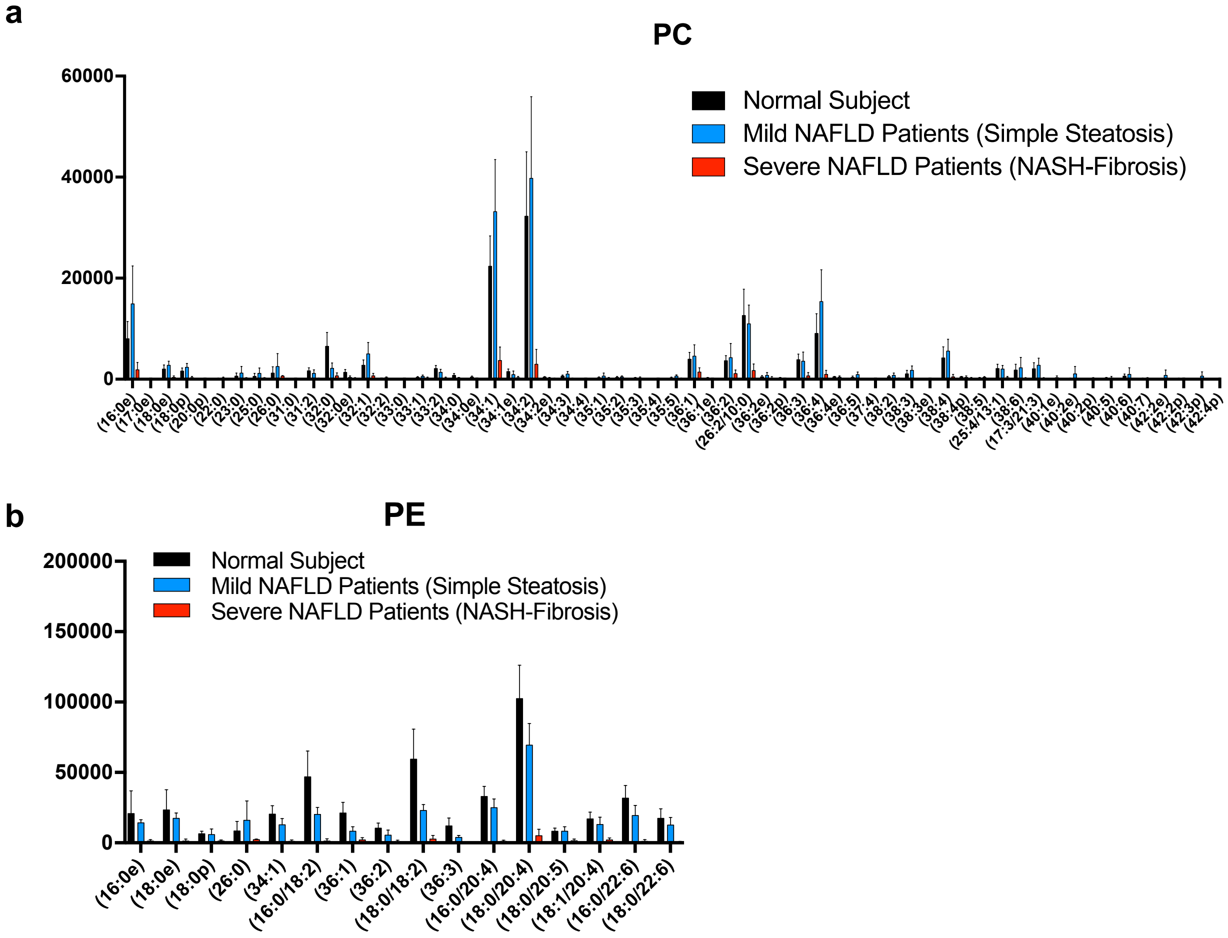

Supplementary Figure 11. Levels of individual PC or PE molecules in liver samples of 15 normal, 15 simple steatosis, and 15 severe NASH-fibrosis patients were determined by LC-MS. Means +/- SD are shown (n=15 human).

Supplementary Figure 12. Full size immunoblots of cropped blots in the main manuscript figures.

a Full size immunoblots of cropped blots for Fig. 4a and 4b

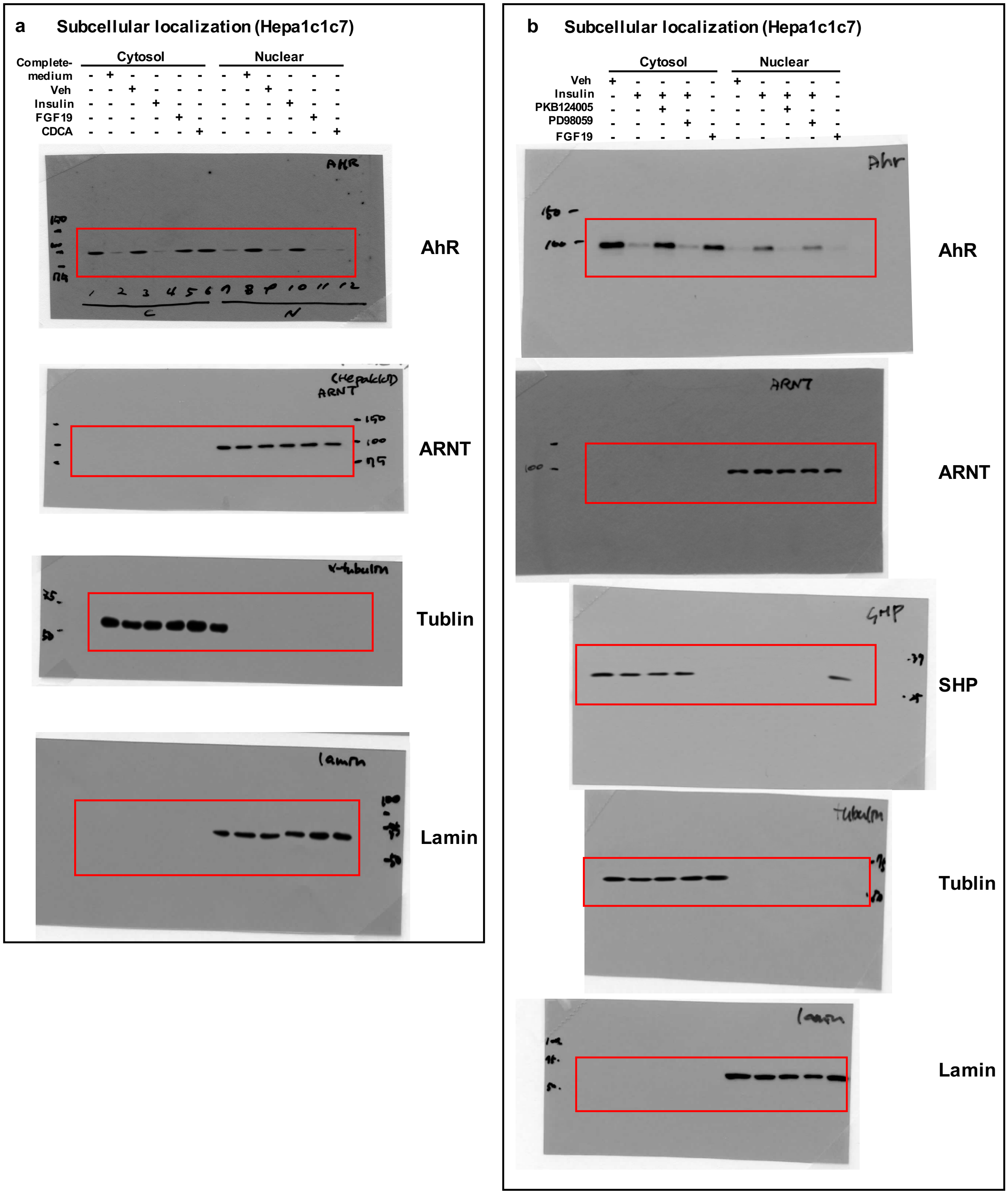

Supplementary Figure 12. Full size immunoblots of cropped blots in the main manuscript figures.

b Full size immunoblots of cropped blots for Fig. 5f

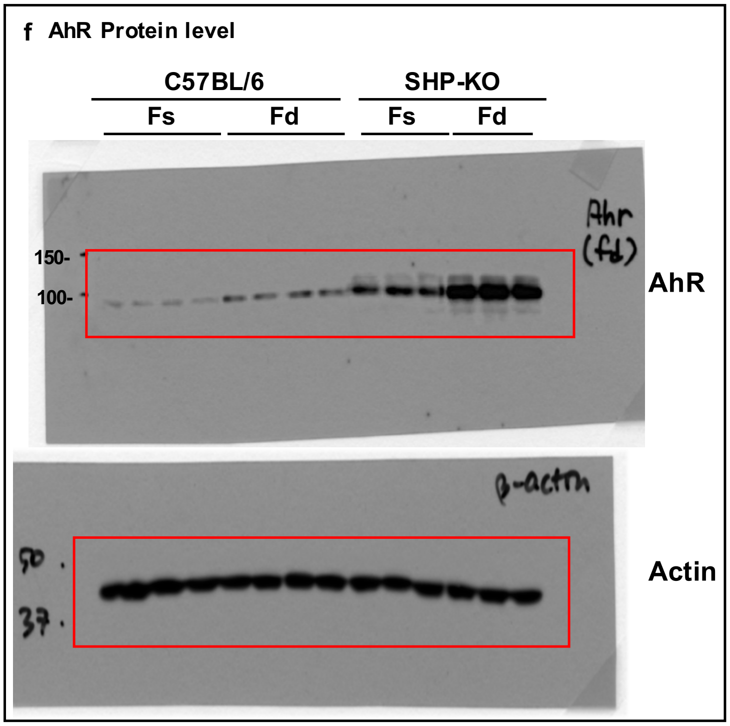

c Full size immunoblots of cropped blots for Fig. 6c

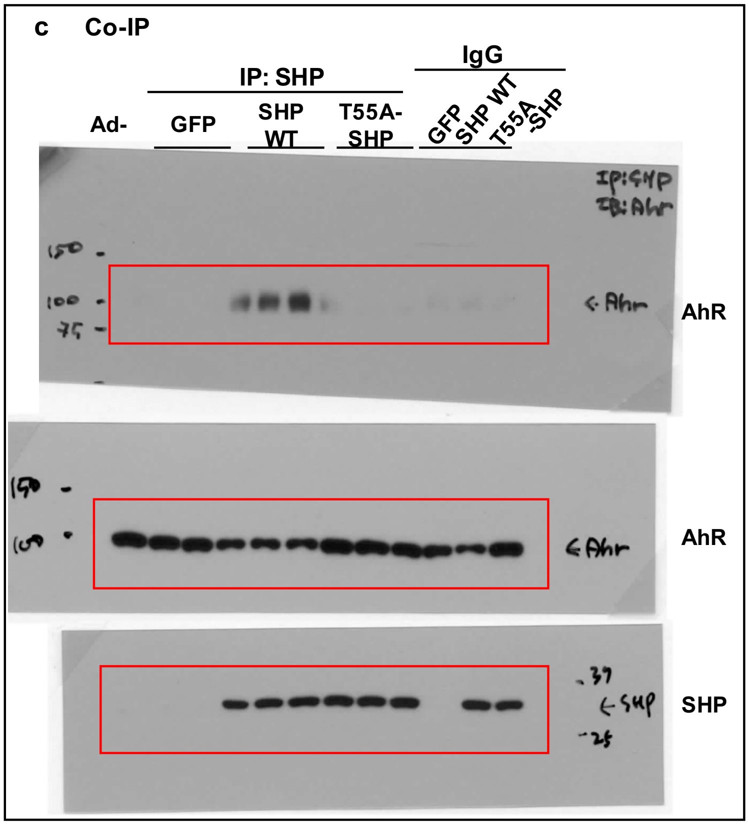

Supplementary Figure 12. Full size immunoblots of cropped blots in the main manuscript figures.

d Full size immunoblots of cropped blots for Fig. 7b

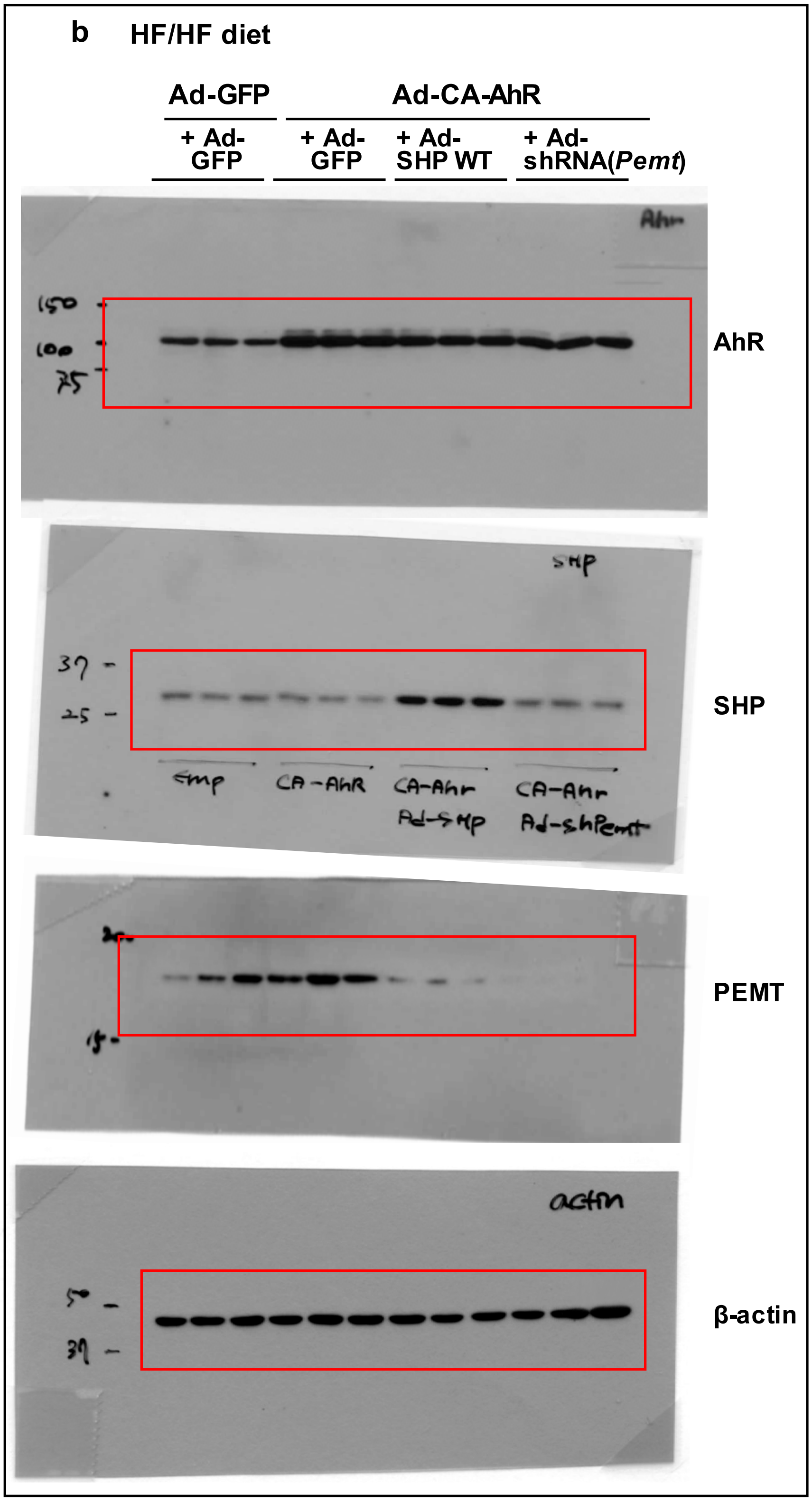

Supplementary Figure 12. Full size immunoblots of cropped blots in the main manuscript figures.

e Full size immunoblots of cropped blots for Fig. 8b

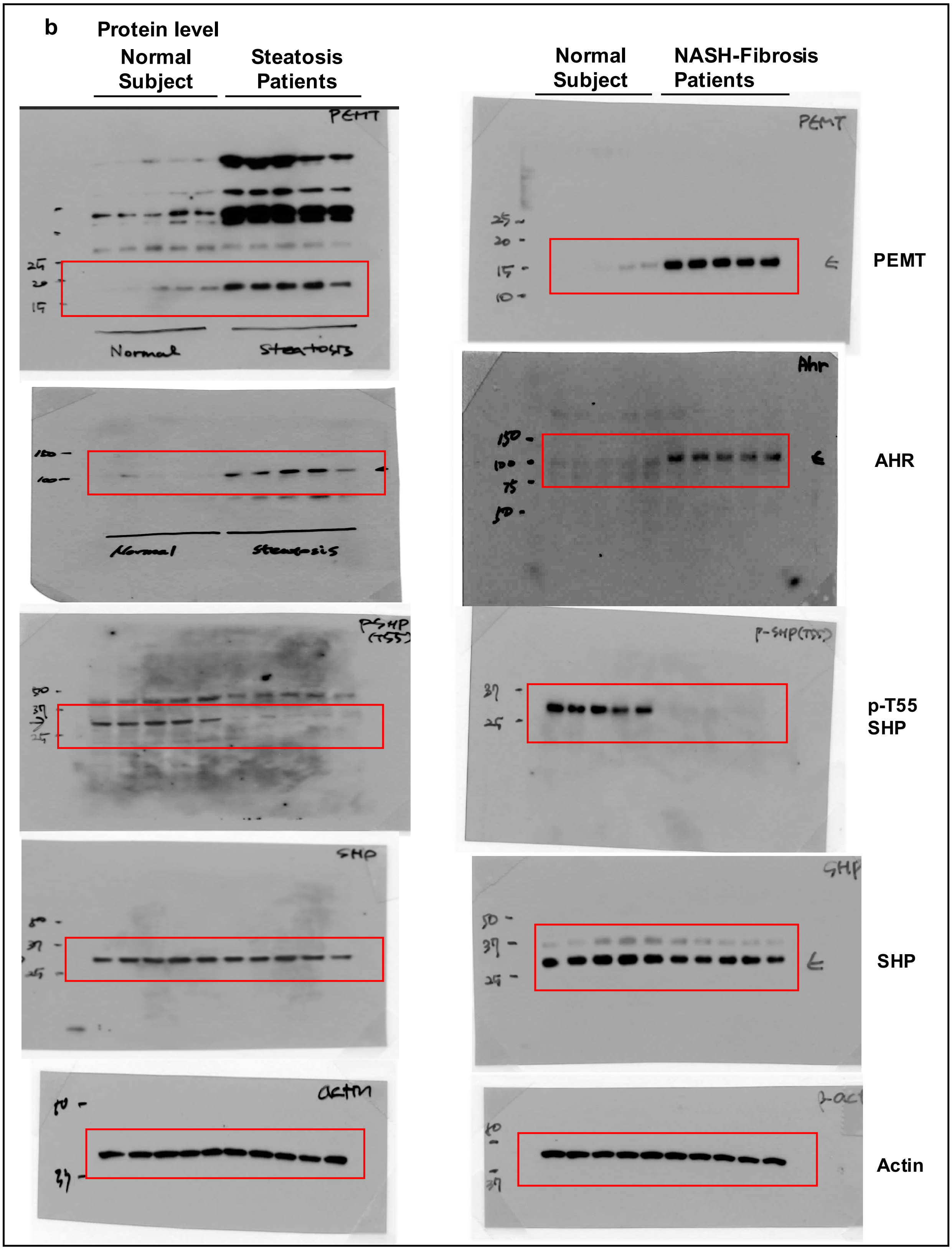

Supplementary Figure 13. Full size immunoblots of cropped blots in the Supplementary figures.

a Full size immunoblots of cropped blots for Supplementary Figure 3a and 3b

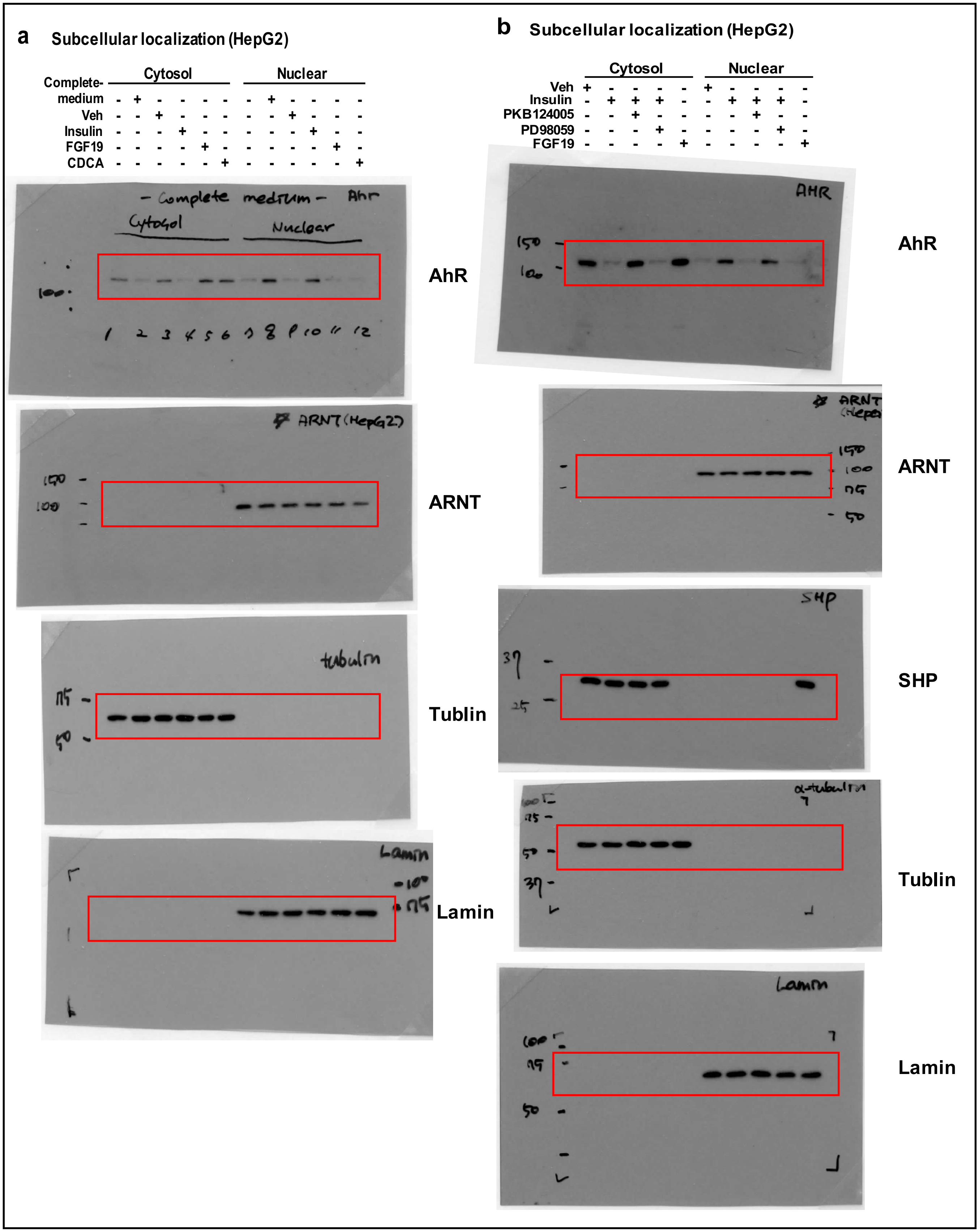

Supplementary Figure 13. Full size immunoblots of cropped blots in the Supplementary figures.

b Full size immunoblots of cropped blots for Supplementary Figure 10

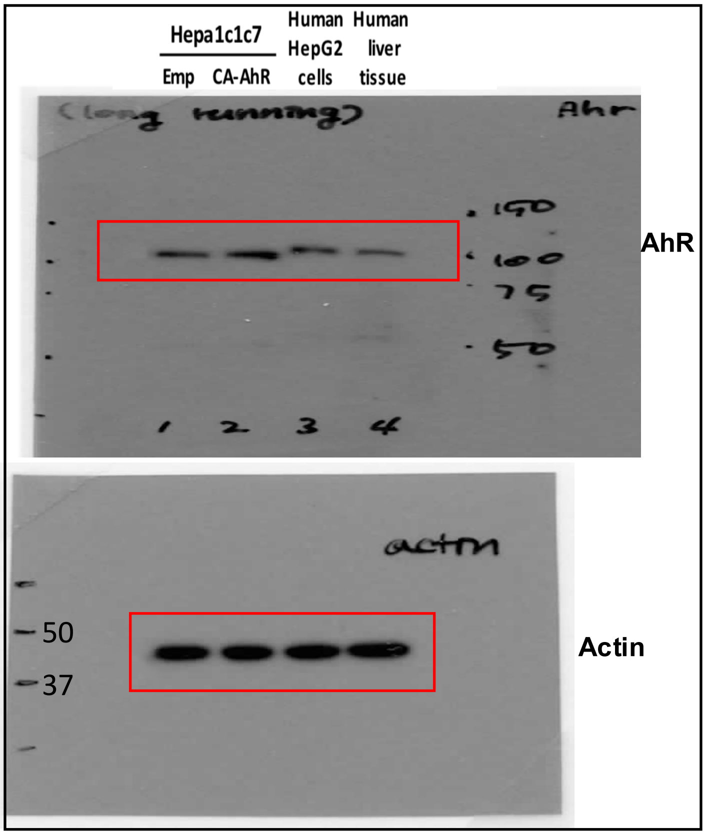

Supplementary Tables

Supplementary Table 1. Sequences of the primers used in ChIP assays

Species	Type	Gene	Sequence
mouse	ChIP	AhR	AGTCCGTCCACCAGTTCG
mouse	ChIP	AhR	TCTTGATGTCTGGGTTACAAGG
mouse	ChIP	Ahcyl2	GGAGGAGGGACGAAAGGAC
mouse	ChIP	Ahcyl2	CAGAACCTGTAGTGCGCTTG
mouse	ChIP	Mthfr	CGCCATCTTCCTCCTTTG
mouse	ChIP	Mthfr	CACATCTACGAGACGAGACGAC
mouse	ChIP	Ahcyl2	GGAGGAGGGACGAAAGGAC
mouse	ChIP	Ahcyl2	CAGAACCTGTAGTGCGCTTG
mouse	ChIP	Atic	CTGTCCGTCCCTTGACTCC
mouse	ChIP	Atic	TTCTCATTACTCGCGGCTTC
mouse	ChIP	Mtfmt	GTGGCTGATGGGAAGGAG
mouse	ChIP	Mtfmt	GGAGATGGAGGGTCTGCTG
mouse	ChIP	Cyp1a1	CGGTTGTGAGTTGGGTAGC
mouse	ChIP	Cyp1a1	TAAGCCTGCTCCATCCTCTG
mouse	ChIP	Gnmt	TCTCCCATGCCCATACTACC
mouse	ChIP	Gnmt	CCGTACCGCAGAGTACAAGG
mouse	ChIP	Pemt	GGAGCTGTACCTGCCTGAAG
mouse	ChIP	Pemt	ATGCCCTAGTCCCTCTCTCC
mouse	ChIP	Aldh1a1	TTTCTTCTCCAGTTGATTTCT
mouse	ChIP	Aldh1a1	CGCTTATATCTATCCTTCTATTCT
mouse	ChIP	Dhfr	GCAAGTGGTACACAGCTCAGG
mouse	ChIP	Dhfr	CCAGGGTAGGTCTCCGTTC

Supplementary Table 2. Sequences of the primers used in q-RTPCR assays

Species	Type	Gene	Sequence
Mouse	mRNA	36B4 (control)	TGCTGAACATGCTCAAC
Mouse	mRNA	36B4 (control)	GTCGAACACCTGCTGGATGAC
Mouse	mRNA	AhR	TCCACAACTGGCTTTGTTTG
Mouse	mRNA	AhR	CCAGAATAAGCTGCCCTTTG
Mouse	mRNA	Mtfmt	CAGACTCTCAGACTTCCA
Mouse	mRNA	Mtfmt	TGTCATCTTCTTGCTTGTAA
Mouse	mRNA	Aldh1a1	TTTCTTCTCCAGTTGATTTCT
Mouse	mRNA	Aldh1a1	CGCTTATATCTATCCTTCTATTCT
Mouse	mRNA	Gnmt	GCTTTCAGGAGATGGCTTTG
Mouse	mRNA	Gnmt	GTGGGCTTTGTTGTTGACTG
Mouse	mRNA	Pemt	AGCAGAGAACTCGGAAGCTG
Mouse	mRNA	Pemt	CCAGGAAGTAGGTGGTGTGG
Mouse	mRNA	Ahcyl2	GTCCCACGGTCTTCTAGTCG
Mouse	mRNA	Ahcyl2	CTGACTCTCGCACCAGCTC
Mouse	mRNA	Atic	CTGTCCGTCCCTTGACTCC
Mouse	mRNA	Atic	TTCTCATTACTCGCGGCTTC
Mouse	mRNA	Mthfr	GTGGTCCTCTGTGCCTCTTC
Mouse	mRNA	Mthfr	AGCCAGCCTCTGCTTAGATG
Mouse	mRNA	Cyp1a1	CAGCATCCTCTTGCTACTTGG
Mouse	mRNA	Cyp1a1	TGAGGCTGTCTGTGATGTCC
Mouse	mRNA	Dhfr	GCTGGGTGCAGTCTTAGGAG
Mouse	mRNA	Dhfr	AGGGAGCACTGAAGAAGTGG
Mouse	pre-mRNA	Pemt	AAATACGAGCCCGACAACTG
Mouse	pre-mRNA	Pemt	ACCACTACCACCACCAATCG
Mouse	pre-mRNA	AhR	TTTCGTCGGTAGAGCAGTCC
Mouse	pre-mRNA	AhR	CTGTGTCGCTTAGAAGGATTTG
Mouse	pre-mRNA	Cyp1a1	TTCCTGTCCTCCGTTACCTG
Mouse	pre-mRNA	Cyp1a1	CCTAACTGCTTCCCATCACC
Human	mRNA	hPEMT	GCTCTCCAGCTTCTTTGCAC
Human	mRNA	hPEMT	AGGTAGTTGGCTGTGCTTCC
Human	mRNA	hGNMT	AGCCAACTGGATGACTCTGG
Human	mRNA	hGNMT	GTCTGGCAAGTGAGCGAAAC
Human	mRNA	hAHR	ACTCCACTTCAGCCACCATC
Human	mRNA	hAHR	CTTCCTTTGGCATCACAACC
Human	mRNA	hSHP	CAGAGATCAGGTGGGCAGAG
Human	mRNA	hSHP	TGTGGCTGAGTGAAGAGCTG

Supplementary Table 3. Sequences in the indicated genes of binding motifs that were identified by Jaspar and summarized in Fig. 3.

Gene	Motifs	predicted site sequence
AhR	Ahr::Arnt	CGCGTG
AhR	Ahr::Arnt	CGCGTG
AhR	Nr1h3::Rxra	TGACGCTCGGTGCCCCACG
AhR	Ahr::Arnt	TGTGTG
AhR	Ahr::Arnt	TGTGTG
AhR	Ahr::Arnt	CGTGTG
AhR	Ahr::Arnt	AGCGTG
AhR	Ahr::Arnt	TGCGCG
AhR	Ahr::Arnt	CACGTG
AhR	Ahr::Arnt	CACGTG
AhR	CREB1	TGACGCAG
AhR	Ahr::Arnt	TGCGTC
AhR	SREBF1	ACCACCCCAC
AhR	SREBF2	GTGGGGTGGT
AhR	FOXA1	GTGGTGTTCGCCCAG
AhR	Foxo1	ACCTGTTTTCC
AhR	SREBF1	CTCACACCCC
AhR	CREB1	TGAGGCCA
AhR	FOXA1	AAGGAATTTGCCTAG
AhR	CREB1	TGATGTCT
Pemt	Model name	predicted site sequence
Pemt	Ahr::Arnt	TGCCTG
Pemt	Foxo1	TCGTATTTTCT
Pemt	SREBF1	ATCACGCGAG
Pemt	SREBF2	CTCGCGTGAT
Pemt	Ahr::Arnt	CGCGTG
Pemt	Ahr::Arnt	CACGTG
Pemt	Ahr::Arnt	CACGTG
Pemt	Ahr::Arnt	CGCATG
Pemt	FOXA1	GGCCTCTTGGCATTT
Pemt	Ahr::Arnt	CTCGTG
Dnmt3a	Model name	predicted site sequence
Dnmt3a	SREBF1	ACCACCCGAG
Dnmt3a	Ahr::Arnt	CGGGTG
Dnmt3a	Pparg::Rxra	GAGGGGCACAGGGCG
Dnmt3a	Ahr::Arnt	CCCGTG
Dnmt3a	Ahr::Arnt	TGCCTG
Ahcyl2	Model name	predicted site sequence
Ahcyl2	SREBF1	ATGACGCGAC
Ahcyl2	SREBF1	GTCGCGTCAT
Ahcyl2	SREBF2	ATGACGCGAC
Ahcyl2	CREB1	TGACGCGA
Ahcyl2	FOXA1	TCCGTTTTTATACTT
Ahcyl2	Nr5a2	AACTCCCAGGACAGC
Ahcyl2	Pparg::Rxra	GTGGGGGATAGGCCG
Ahcyl2	Ahr::Arnt	TGGGTG
Ahcyl2	Nr1h3::Rxra	TGCACTGCGCTCGCCTCGC
Ahcyl2	YY1	CAAGTTGGTTGC
Ahcyl2	FOXA1	AAGTTGGTTGCTGAG
Ahcyl2	CREB1	TGAGGTCC
Ahcyl2	Ahr::Arnt	CGCGTG
Ahcyl2	Ahr::Arnt	CGCTTG
Ahcyl2	Ahr::Arnt	CGCGTG
Ahcyl2	SREBF1	CTCGCCCCAC
Ahcyl2	SREBF2	GTGGGGCGAG
Ahcyl2	Ahr::Arnt	CGAGTG
Ahcyl2	Ahr::Arnt	TGCGAG
Ahcyl2	CREB1	CGACATCA
Ahcyl2	CREB1	TGACACAA
Ahcyl2	FOXA1	CCGCTGCTGACACAA
Ahcyl2	Ahr::Arnt	TGCCTG
Ahcyl2	YY1	TACCATGGCGCC
Ahcyl2	Ahr::Arnt	CGGGTG
Gnmt	Ahr::Arnt	TGGGTG
Gnmt	FOXA1	GCAATATCTGCATTA
Gnmt	Pparg::Rxra	TTAGGGTAGGGGCCA
Gnmt	SREBF1	CTCAGGCAAT
Gnmt	Ahr::Arnt	TGCCTG
Gnmt	Nr1h3::Rxra	GGGCTTGAAGTCACCCAGG
Gnmt	CREB1	TGACTTCA
Gnmt	CREB1	TGAAGTCA
Gnmt	Ahr::Arnt	TGGGTG
Gnmt	Ahr::Arnt	TGCGAG
Gnmt	Ahr::Arnt	TCCGTG
Gnmt	CREB1	AGACGCCA
Gnmt	YY1	CAACAGGGCCCC
Gnmt	CREB1	TGAGGCCA
Gnmt	Nr1h3::Rxra	TGGCCTCAGGTCTCCCCAC
Gnmt	SREBF1	GTCTCCCCAC
Gnmt	SREBF2	GTGGGGAGAC
Gnmt	Pparg::Rxra	GCAGGAGAGAGGTGG
Gnmt	SREBF1	ACCACGTCAT
Gnmt	SREBF2	ATGACGTGGT
Gnmt	CREB1	TGACGTGG
Gnmt	CREB1	CCACGTCA
Gnmt	CREB1	TGAAGCCA

Gene	Gene	Motifs	predicted site sequence
AhR	Mat2a	Ahr::Arnt	CTCGTG
AhR	Mat2a	Pparg::Rxra	GAAGGGCGGAGGGCA
AhR	Mat2a	Ahr::Arnt	TGGGTG
AhR	Mat2a	Nr5a2	TAGGGGAAGGTCACC
AhR	Mat2a	Ahr::Arnt	CTCGTG
AhR	Mat2a	SREBF1	GTCTCCTCAG
AhR	Mat2a	SREBF2	CTGAGGAGAC
AhR	Mat1a	Ahr::Arnt	TGCGTG
AhR	Mat1a	YY1	CGAAATGGCCGT
AhR	Mat1a	Nr1h3::Rxra	TGTCCCATACTCACCTCTT
AhR	Mat1a	SREBF1	CTCACCTCTT
AhR	Mat1a	Nr1h3::Rxra	TCTCCCAGAGTGCCCTCCT
AhR	Mat1a	Pparg::Rxra	TGAGGGTAGAAGGCA
AhR	Mat1a	Nr5a2	CAGGGCAAAGCCAGC
AhR	Mat1a	FOXA1	AAGATGTTTGCAGAT
AhR	Mat1a	SREBF1	GTCATGTCAT
AhR	Mat1a	SREBF2	ATGACATGAC
AhR	Mat1a	CREB1	TGACATGA
AhR	Mat1a	Ahr::Arnt	TGCCTG
AhR	Mat1a	SREBF1	ACCAGGCCAT
Pemt	Mat1a	SREBF2	ATGGCCTGGT
Pemt	Mat1a	YY1	TAGAATGGAGGA
Pemt	Mat1a	FOXA1	TAGATATGTACCCAG
Pemt	Mat1a	FOXA1	CATCTGGGTACATAT
Pemt	Mat1a	Pparg::Rxra	GGAAGCCAGAGGTCT
Pemt	Mat1a	YY1	AAATATGGTCAA
Pemt	Mat1a	SREBF1	CTCACACGAT
Pemt	Mat1a	SREBF2	ATCGTGTGAG
Pemt	Mat1a	Ahr::Arnt	CGTGTG
Pemt	Mat1a	FOXA1	GAGCTGTGTGTTCTT
Pemt	Mat1a	Ahr::Arnt	TGTGTG
Dnmt3a	Mat1a	FOXA1	AAGGTGTTGTCTTTA
Dnmt3a	Mat1a	Foxo1	AGGTGTTGTCT
Dnmt3a	Mat1a	FOXA1	TTACTGTGGATAATA
Dnmt3a	Mat1a	FOXA1	CATTTCTTGGCTCTG
Dnmt3a	Mat1a	YY1	CAAGATGTTGAC
Dnmt3a	Mat1a	Foxo1	AGATGTTGACC
Ahcyl2	Mat1a	CREB1	TGAGGCTA
Ahcyl2	Mat1a	FOXA1	TGGCTACTTATTTAA
Ahcyl2	Mat1a	Nr1h3::Rxra	TGGCTCTTTGTGAGCCCTG
Ahcyl2	Mat1a	FOXA1	TCCATTTTGATTTTG
Ahcyl2	Mat1a	YY1	CAAAATGGAGAC
Ahcyl2	Mat1a	Ahr::Arnt	TGTGTG
Ahcyl2	Mat1a	Ahr::Arnt	TGCTTG
Ahcyl2	Mat1a	Foxo1	ACATGTTTCTA
Ahcyl2	Mat1a	SREBF1	CTCACAGCAT
Ahcyl2	Mat1a	FOXA1	TGCATCTGTGCCTTG
Ahcyl2	Mat1a	Pparg::Rxra	CTGGGGGAAAGGGGA
Ahcyl2	Mat1a	Ahr::Arnt	AGCGTG
Ahcyl2	Mat1a	FOXA1	CTGCTGTTTCCTCAC
Ahcyl2	Mat1a	Foxo1	TGCTGTTTCCT
Ahcyl2	Mat1a	FOXA1	GATCTGTTGGCCAAT
Ahcyl2	Mat1a	Ahr::Arnt	TGTGTG
Ahcyl2	Mat1a	Ahr::Arnt	TGTGTG
Ahcyl2	Mat1a	Ahr::Arnt	TGCCTG
Ahcyl2	Dhfr	Ahr::Arnt	TCCGTG
Ahcyl2	Dhfr	Ahr::Arnt	TGCGGG
Ahcyl2	Dhfr	Ahr::Arnt	CGCCTG
Ahcyl2	Dhfr	Ahr::Arnt	TGCGCG
Ahcyl2	Dhfr	Ahr::Arnt	TGCGCG
Ahcyl2	Dhfr	Ahr::Arnt	GGCGTG
Ahcyl2	Dhfr	Ahr::Arnt	TGCGGG
Ahcyl2	Dhfr	Nr5a2	GCCCCCAAGGCCGGT
Gnmt	Dhfr	FOXA1	TATTTGTGTAGCTAA
Gnmt	Dhfr	Ahr::Arnt	TGCGCG
Gnmt	Dhfr	Ahr::Arnt	TGCGCG
Gnmt	Dhfr	Ahr::Arnt	TGCGCG
Gnmt	Dhfr	Foxo1	AGCTGTGTACC
Gnmt	Dhfr	CREB1	TGCCGTCA
Gnmt	Dhfr	CREB1	TGACGGCA
Gnmt	Dhfr	Ahr::Arnt	AGCGTG
Gnmt	Dhfr	YY1	CATGATGGCAGC
Gnmt			
Gnmt			
Gnmt			
Gnmt			
Gnmt			
Gnmt			
Gnmt			
Gnmt			
Gnmt			
Gnmt			
Gnmt			
Gnmt			
Gnmt			
Gnmt			

Gene	Motifs	predicted site sequence
Mthfr	Ahr::Arnt	CGTGTG
Mthfr	Ahr::Arnt	TCCGTG
Mthfr	YY1	GAAGATGGCGGG
Mthfr	Ahr::Arnt	TGGGTG
Mthfr	SREBF1	GTCACGTGAG
Mthfr	SREBF1	CTCACGTGAC
Mthfr	SREBF2	CTCACGTGAC
Mthfr	CREB1	TCACGTGA
Mthfr	CREB1	TCACGTGA
Mthfr	Ahr::Arnt	CACGTG
Mthfr	Ahr::Arnt	CACGTG
Mthfr	Ahr::Arnt	CACGTG
Mthfr	Ahr::Arnt	CACGTG
Mthfr	Ahr::Arnt	CGGGTG
Mthfr	SREBF1	CTCGCCCCAC
Mthfr	SREBF2	GTGGGGCGAG
Mthfr	SREBF1	CTCGCGTCAC
Mthfr	CREB1	TGACGCGA
Mthfr	SREBF1	GTCACATGAC
Mthfr	SREBF2	GTCATGTGAC
Mthfr	CREB1	TGACGCAG
Mthfr	Ahr::Arnt	TGCGTC
Mthfr	YY1	CAACATGGCCGC
Mthfr	Ahr::Arnt	CGCCTG
Mthfr	FOXA1	GTCCTGTTTCCCTTA
Mthfr	Foxo1	TCCTGTTTCCC
Mthfr	CREB1	TGACGCAA
Mthfr	Ahr::Arnt	TGCGTC
Mthfr	Ahr::Arnt	TGCGTT
Mthfr	SREBF1	AACACACCAC
Mthfr	SREBF2	GTGGTGTGTT
Mthfr	Ahr::Arnt	CGCCTG
Mthfr	Ahr::Arnt	CACGTG
Mthfr	Ahr::Arnt	CACGTG
Mtfmt	SREBF1	ATCACCTCGG
Mtfmt	SREBF2	CCGAGGTGAT
Mtfmt	CREB1	GGAGGCCA
Mtfmt	Ahr::Arnt	AGCGTG
Mtfmt	Ahr::Arnt	TGCTTG
Mtfmt	Ahr::Arnt	CGGGTG
Mtfmt	Ahr::Arnt	TGCGGG
Mtfmt	Ahr::Arnt	GGCGTG
Mtfmt	Ahr::Arnt	GGCGTG
Mtfmt	Nr1h3::Rxra	TGCCCTGGGGTCGCCACCG
Mtfmt	YY1	CAACAGGGCGCC
Atic	Ahr::Arnt	GGCGTG
Atic	Nr1h3::Rxra	CGACCCCACGTGACCCCGA
Atic	Ahr::Arnt	CACGTG
Atic	Ahr::Arnt	CACGTG
Atic	Ahr::Arnt	GGCGTG
Atic	Ahr::Arnt	TGCCTG
Atic	Ahr::Arnt	TGCGTA
Atic	Ahr::Arnt	CACGTG
Atic	Ahr::Arnt	CACGTG
Atic	Ahr::Arnt	TGCGTT
Atic	YY1	CAGCATGGCTCC
Atic	Ahr::Arnt	TGCCTG
Atic	Ahr::Arnt	TGGGTG
Atic	Ahr::Arnt	TGAGTG
Atic	Ahr::Arnt	TGGGTG
Aldh1l1	Ahr::Arnt	TGGGTG
Aldh1l1	Ahr::Arnt	TGGGTG
Aldh1l1	YY1	CAAGATAGCACC
Aldh1l1	CREB1	TGAAGCCA
Aldh1l1	Nr1h3::Rxra	TGTACCCTGTTGACCTCAG
Aldh1l1	FOXA1	ACCCTGTTGACCTCA
Aldh1l1	Foxo1	CCCTGTTGACC
Aldh1l1	CREB1	TGAGGTCA
Aldh1l1	CREB1	TGACCTCA
Aldh1l1	FOXA1	TGTCTGGTGACTTAG
Aldh1l1	SREBF1	GTCACCAGAC
Aldh1l1	Nr5a2	GGGGTCCAGGCCAAG
Aldh1l1	Foxo1	TGCTGGTGACA
